# Synaptic proteasome is inhibited in Alzheimer’s disease models and associates with memory impairment in mice

**DOI:** 10.1038/s42003-023-05511-9

**Published:** 2023-11-07

**Authors:** Felipe C. Ribeiro, Danielle Cozachenco, Luana Heimfarth, Juliana T. S. Fortuna, Guilherme B. de Freitas, Jorge M. de Sousa, Soniza V. Alves-Leon, Renata E. P. Leite, Claudia K. Suemoto, Lea T. Grinberg, Fernanda G. De Felice, Mychael V. Lourenco, Sergio T. Ferreira

**Affiliations:** 1 Institute of Medical Biochemistry Leopoldo de Meis, Federal University of Rio de Janeiro, Rio de Janeiro, RJ Brazil; 2 Institute of Biomedical Sciences, Federal University of Rio de Janeiro, Rio de Janeiro, RJ Brazil; 3 Centre for Neuroscience Studies, Department of Biomedical and Molecular Sciences and Department of Psychiatry, Queen’s University, Kingston, ON Canada; 4 Division of Neurosurgery, Clementino Chagas Filho University Hospital, Federal University of Rio de Janeiro, Rio de Janeiro, RJ Brazil; 5 Division of Neurology, Clementino Chagas Filho University Hospital, Federal University of Rio de Janeiro, Rio de Janeiro, RJ Brazil; 6 Translational Neuroscience Laboratory, Federal University of the State of Rio de Janeiro, Rio de Janeiro, RJ Brazil; 7 Department of Pathology, University of São Paulo Medical School, São Paulo, SP Brazil; 8 Department of Neurology, Memory and Aging Center, University of California, San Francisco, CA USA; 9 D’Or Institute for Research and Education, Rio de Janeiro, RJ Brazil; 10 Institute of Biophysics Carlos Chagas Filho, Federal University of Rio de Janeiro, Rio de Janeiro, RJ Brazil

**Keywords:** Molecular neuroscience, Cellular neuroscience

## Abstract

The proteasome plays key roles in synaptic plasticity and memory by regulating protein turnover, quality control, and elimination of oxidized/misfolded proteins. Here, we investigate proteasome function and localization at synapses in Alzheimer’s disease (AD) *post-mortem* brain tissue and in experimental models. We found a marked increase in ubiquitinylated proteins in *post-mortem* AD hippocampi compared to controls. Using several experimental models, we show that amyloid-β oligomers (AβOs) inhibit synaptic proteasome activity and trigger a reduction in synaptic proteasome content. We further show proteasome inhibition specifically in hippocampal synaptic fractions derived from APPswePS1ΔE9 mice. Reduced synaptic proteasome activity instigated by AβOs is corrected by treatment with rolipram, a phosphodiesterase-4 inhibitor, in mice. Results further show that dynein inhibition blocks AβO-induced reduction in dendritic proteasome content in hippocampal neurons. Finally, proteasome inhibition induces AD-like pathological features, including reactive oxygen species and dendritic spine loss in hippocampal neurons, inhibition of hippocampal mRNA translation, and memory impairment in mice. Results suggest that proteasome inhibition may contribute to synaptic and memory deficits in AD.

## Abstract (web summary)

A study involving several research models suggests that the function and synaptic localization of proteasomes, intracellular machineries involved in protein degradation, are impaired in the brains affected by Alzheimer’s disease.

## Introduction

The ubiquitin-proteasome system (UPS) is the main cellular machinery for protein degradation. Proteins targeted for proteasomal degradation are tagged by lysine 48 (K48)-polyubiquitination for recognition by the 26S proteasome^1^. The 26S proteasome is comprised of a core 20S catalytic particle and one or two 19S regulatory particles^1^. The 20S particle exhibits proteolytic activity and is well known for its role in the degradation of damaged, misfolded, and intrinsically disordered proteins, while association with 19S particles confers specificity for protein degradation^2^.

Protein degradation by the proteasome mediates synaptic plasticity and memory processes. Inhibiting brain proteasome activity impairs synaptogenesis^3^, the maintenance of long-term potentiation^4^, and both the formation and extinction of memories in mice^5–7^. Evidence indicates that neuronal activity promotes calcium-calmodulin-dependent kinase IIα (CaMKIIα)-mediated recruitment and activity of proteasomes at synapses, and that this is necessary for long-lasting changes in synapse structure and strength^8–10^.

Alzheimer’s disease (AD) is characterized by synapse dysfunction and memory deficits^11,12^. Early studies reported that proteasome activity was inhibited in AD brains^13,14^ and by the amyloid-β peptide (Aβ) in cell-free assays^15,16^, and by intraneuronal accumulation of Aβ in cellular models^17^. Altered proteasome localization and accumulation of polyubiquitinylated substrates has been associated with synaptic presence of tau oligomers in AD brains^18^. Given the role of proteasomes in synaptic plasticity, impaired proteasome localization and activity might impact synapse function and contribute to memory impairment in AD^19^. Here, we investigated proteasome activity and synaptic localization in *postmortem* AD brain and in several AD models, including human ex vivo cortical tissue, primary neuronal cultures and mouse models, and determined the impact of proteasome inhibition on memory in mice.

## Results

### Proteasome inhibition in postmortem AD hippocampi and in ex vivo human cortical tissue

**Fig. 1:**
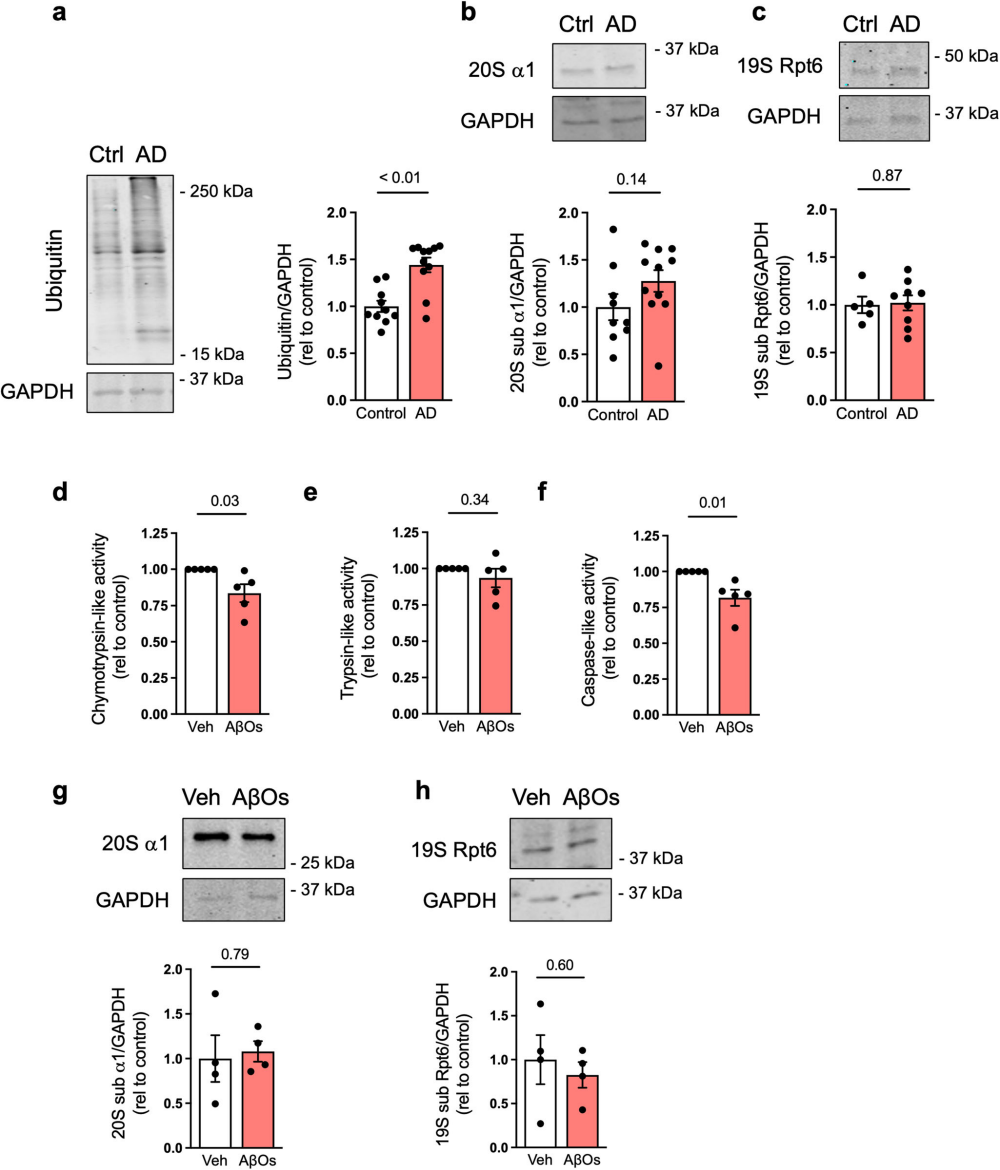
Ubiquitinylated proteins accumulate in AD brains, and proteasome activity is impaired in human cortical slices exposed to AβOs in culture. Immunoreactivities of polyubiquitin (*n* = 10 control, 11 AD) (**a**), 20S subunit α1 (*n* = 9 control, 11 AD) (**b**) and 19S subunit Rpt6 (*n* = 5 control, 9 AD) (**c**) were quantified in AD hippocampi compared to controls; two-tailed unpaired Student’s *t* test. **d**–**f** Chymotrypsin-, trypsin- and caspase-like proteasome activities in human adult cortical slices in culture exposed to vehicle or 0.5 µM AβOs for 24 h (*n* = 5 independent donors; two-tailed unpaired Student’s *t* test). **g**, **h** Proteasome 20S subunit α1 and 19S subunit Rpt6 immunoreactivities in human cortical slices in culture. GAPDH was used as loading control (*n* = 4 independent donors; two-tailed unpaired Student’s *t* test). Data are presented as mean ± SEM.

We initially examined whether *postmortem* brain tissue from AD and age-matched non-cognitively impaired subjects would differ in terms of polyubiquitinylated protein content as a proxy of proteasome activity. We found a marked increase in ubiquitinylated proteins in AD hippocampi compared to controls (Fig. 1a and Supplementary Data 1). No changes were observed in total immunoreactivities of α1 and Rpt6, protein markers of 20S and 19S proteasome subunits, respectively (Fig. 1b, c). The accumulation of ubiquitinylated proteins in the absence of altered 20S or 19S proteasome subunits suggests that proteosome activity is impaired in the AD hippocampus.

Considerable evidence indicates that Aβ oligomers (AβOs) accumulate in AD brains, trigger synapse damage and cognitive defects in mice, and correlate with cognitive impairment in humans (reviewed in refs. ^11,20^). To investigate the impact of AβOs on proteasome activity in human brain tissue, we exposed human adult cortical slices in culture to exogenously added AβOs (0.5 µM) for 24 h. Chymotrypsin- and caspase-like activities of the proteasome were reduced in AβO-exposed cortical slices, compared to slices exposed to vehicle (Fig. 1d–f). Consistent with our observations in *postmortem* AD brain, total immunoreactivities of 20S α1 and 19S Rpt6 proteins were unchanged in human cortical slices exposed to AβOs (Fig. 1g, h).

### AβOs trigger proteasome inhibition and mislocalization in primary hippocampal neurons

**Fig. 2:**
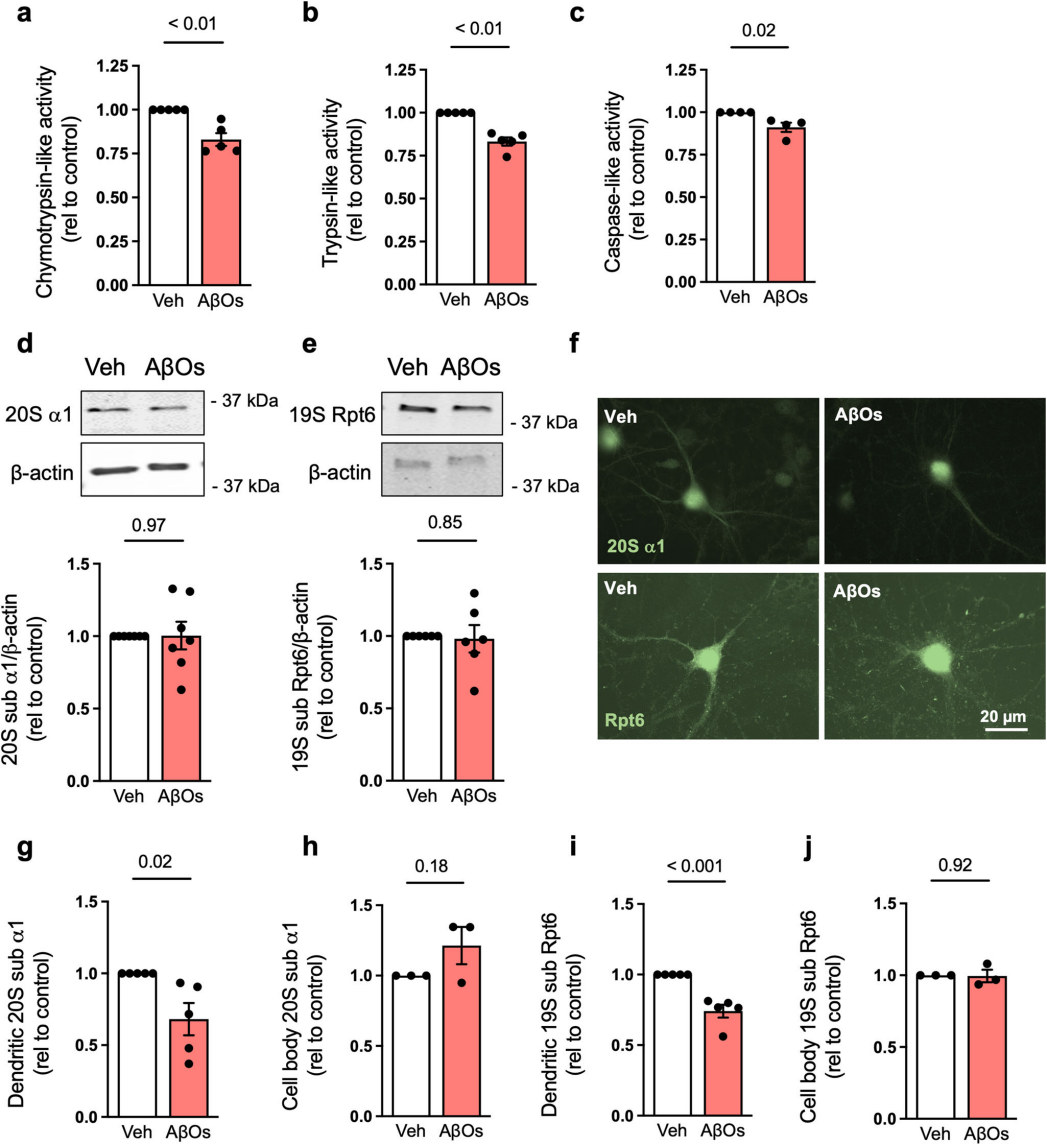
AβOs induce proteasome inhibition in hippocampal cultures. **a**–**c** Chymotrypsin- (*n* = 5), trypsin- (*n* = 5) and caspase-like (*n* = 4) proteasome activities in primary hippocampal cultures exposed to vehicle or 0.5 µM AβOs for 24 h (*n* = 4–5 independent cultures; two-tailed unpaired Student’s *t* test). **d**, **e** Proteasome 20S subunit α1 (*n* = 7) and 19S subunit Rpt6 (*n* = 6) in hippocampal cultures exposed to vehicle or 0.5 µM AβOs (*n* = 6–7 independent cultures; two-tailed unpaired Student’s *t* test). **f**–**j** Primary hippocampal cultures were exposed to vehicle or 0.5 µM AβOs for 24 h and were then labeled for proteasome 20S subunit α1 or 19S subunit Rpt6 (**f**). Quantification of dendritic (**g**, **i**) or cell body (**h**, **j**) immunoreactivities (*n* = 3 for cell body and 5 for dendrites in 20S subunit α1 and 3 for cell body and 5 for dendrites in Rpt6; symbols represent means from 30 images per experimental condition per culture; two-tailed unpaired Student’s *t* test). Data are presented as mean ± SEM. Scale bar: 20 µm.

To determine the impact of AβOs on neuronal proteasome activity, we exposed primary rat hippocampal cultures to AβOs (0.5 µM) for 24 h. Exposure to AβOs inhibited chymotrypsin-, trypsin- and caspase-like activities of the proteasome (Fig. 2a–c and Supplementary Fig. 1a–c). Similar to our findings with human brain tissue, contents of 20S α1 and 19S Rpt6 proteasomal proteins were unchanged in cell homogenates from cultures exposed to AβOs (Fig. 2d, e).

Because the subcellular localization of proteasomes at synapses has been implicated in synaptic plasticity and neuronal function^21,22^, we next investigated whether AβOs would induce changes in synaptic localization of proteasomes. Immunofluorescence labeling in cultured hippocampal neurons showed that exposure to AβOs significantly reduced dendritic levels of α1 and Rpt6, while the immunoreactivities of both proteins in the cell body were unchanged (Fig. 2f–j). These results suggest that AβOs promote proteasome mislocalization in hippocampal neurons, with reduced localization of proteasomes to synaptic sites.

**Fig. 3:**
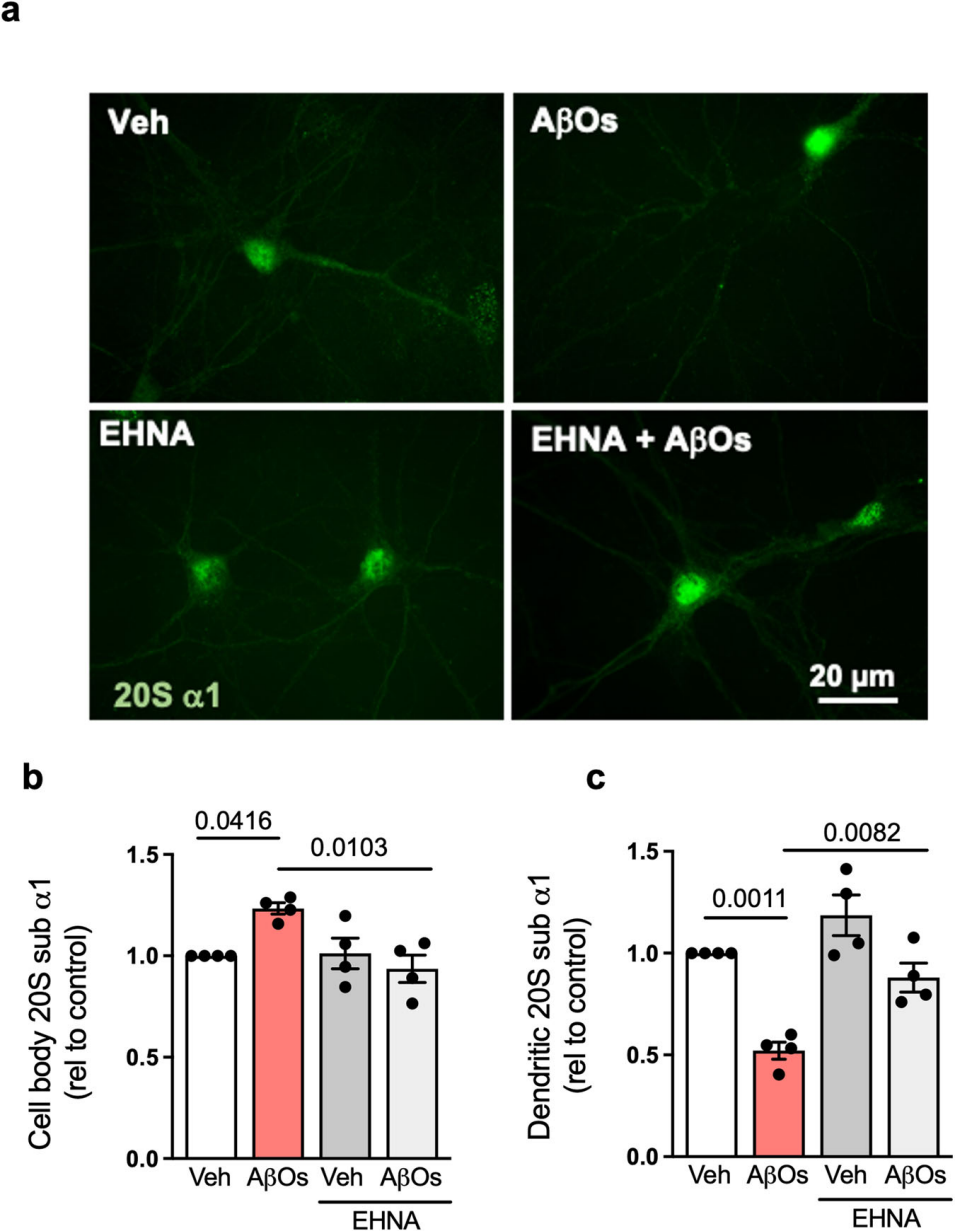
Dynein inhibition prevents AβO-induced synaptic proteasome mislocalization in hippocampal cultures. **a**–**c** Primary hippocampal cultures were exposed to vehicle or 0.5 µM AβOs for 24 h in the presence or absence of erythro-9-[3-(2-hydroxynonyl)]adenine (EHNA; 100 µM), and were then immunolabeled for proteasome 20S subunit α1 (**a**). Quantification of dendritic (**b**) or cell body (**c**) immunoreactivities (*n* = 4 independent cultures; two-tailed two-way ANOVA with Holm-Sidak post hoc test). Data are presented as mean ± SEM. Scale bar: 20 µm.

To investigate the mechanism underlying AβO-induced reduction in synaptic proteasome content, we exposed cultures to AβOs in the presence or absence of erythro-9-[3-(2-hydroxynonyl)]adenine (EHNA), a selective dynein inhibitor^23^. While EHNA alone did not cause changes in proteasome localization, it prevented the reduction in dendritic proteasome content and accumulation of 20S proteasomes in the cell body instigated by AβOs (Fig. 3a–c). Result thus suggest that dynein-dependent mechanisms are implicated in transport of proteasomes from dendritic compartments in the presence of AβOs.

### AβOs inhibit synaptic proteasomes in vivo

**Fig. 4:**
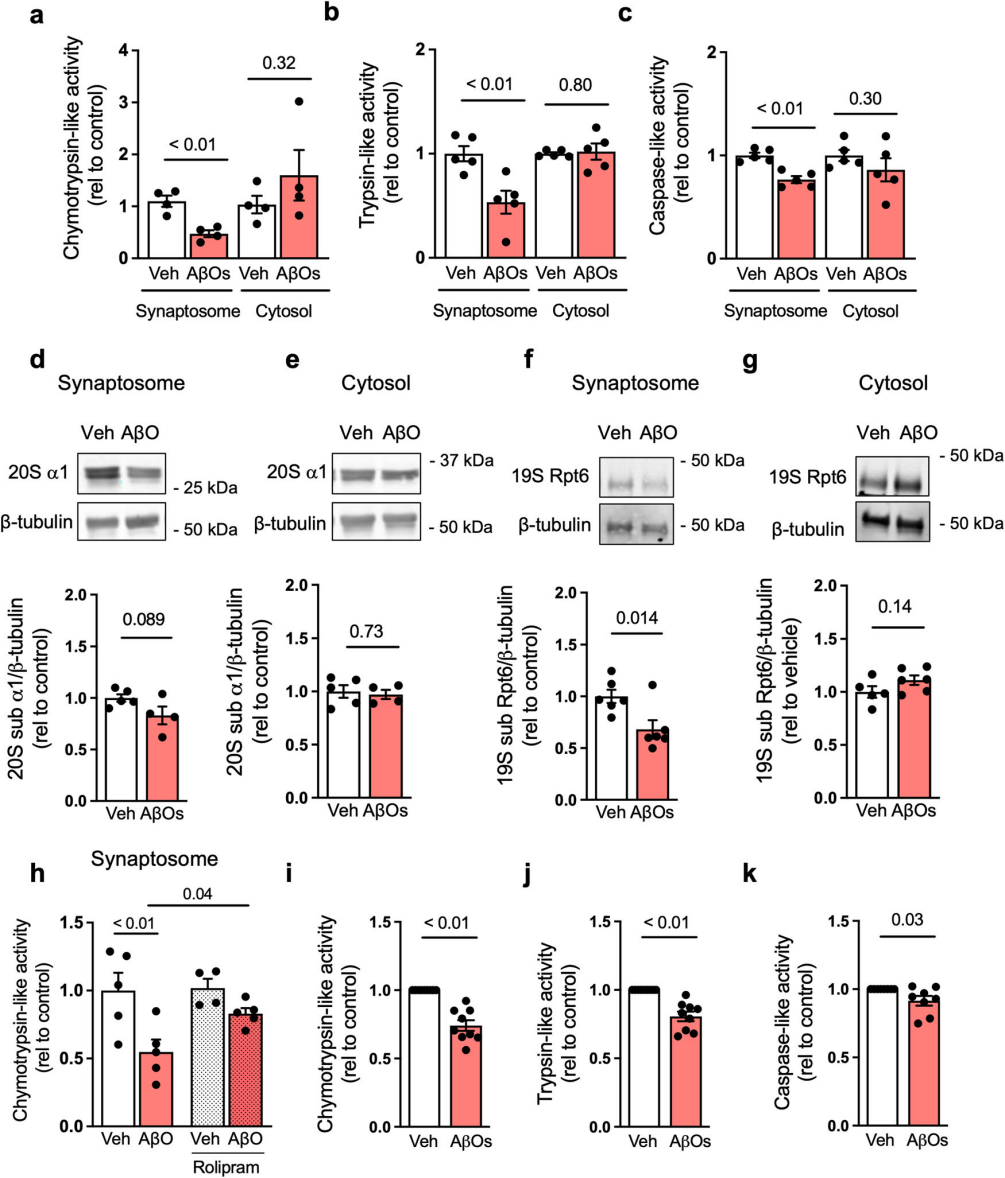
AβOs inhibit synaptic proteasome activity in the mouse hippocampus. Three-month-old Swiss mice received intracerebroventricular (i.c.v.) infusions of 10 pmol AβOs (or vehicle). **a**–**c** Hippocampi were harvested 7 days after infusion, and tissue was fractionated for synaptosome preparation (see “Methods”). Proteasomal chymotrypsin-, (*n* = 4 per group) trypsin- (*n* = 5 per group), and caspase-like activities (*n* = 5 per group) were measured in synaptosomal preparations from independent mice; two-tailed unpaired Student’s *t* test). Proteasome 20S subunit α1 (**d**, **e**) (*n* = 5 veh and 4 AβOs in synaptosomal fraction and *n* = 5 vehicle and 4 AβOs in cytosol fraction) and 19S subunit Rpt6 (**f**, **g**) (*n* = 6 vehicle and 5 AβOs in synaptosomal fraction, 5 in cytosol vehicle and 5 in cytosol AβOs fractions) were determined by Western blotting in synaptosome or cytosolic fractions. **h** Proteasomal chymotrypsin-like activity was measured in synaptosomal preparations from veh-, AβO- and/or rolipram-treated mice (*n* = 5 mice in vehicle, AβOs and rolipram + AβOs; 4 mice in rolipram; two-way ANOVA with Holm-Sidak post hoc test). **i**–**k** Hippocampi from naive mice were harvested and synaptosomes were isolated. Synaptosome preparations were then exposed to AβOs (or vehicle) for 1 h at 37 °C, and proteasomal chymotrypsin-, trypsin-, and caspase-like activities were measured (*n* = 9 synaptosomal preparations from independent mice in chymotrypsin and trypsin, 8 synaptosomal preparations from independent mice for caspase; two-tailed unpaired Student’s *t* test). Data are presented as mean ± SEM.

We next sought to determine the impact of AβOs on proteasome activity and synaptic content in vivo. We performed intracerebroventricular (i.c.v.) infusions of AβOs (10 pmol) in mice and determined proteasome activity and subunit content in hippocampal synaptosomes isolated 7 days after AβO infusion. We found significant reductions in chymotrypsin-, trypsin- and caspase-like proteasome activities in the synaptosome fraction, but not in the cytosol, from the hippocampi of AβO-infused mice (Fig. 4a–c and Supplementary Fig. 1d–f). Furthermore, AβOs instigated reductions in 20S α1 and 19S Rpt6 proteins specifically in hippocampal synaptosomes, but not in the cytosolic fraction (Fig. 4d–g). These results indicate that AβOs induce hippocampal synaptic proteasome inhibition and mislocalization in vivo.

A previous study reported that mutant tau (P301L) accumulation inhibited proteasomes in mice, and that stimulating cAMP/PKA signaling restored brain proteasome activity and memory^24,25^. We thus reasoned that stimulation of cAMP/PKA signaling with rolipram, a phosphodiesterase 4 (PDE4) inhibitor that causes cAMP accumulation^25^, might correct proteasome inhibition by AβOs. To assess this potential mechanism, we treated mice with rolipram (0.1 mg/kg s.c.) for 10 days, and infused AβOs or vehicle (10 pmol via i.c.v.) on the 3rd day after beginning rolipram treatment. We then isolated synaptosomal fractions from the mouse hippocampus and measured proteasomal chymotrypsin activity. We found that rolipram prevented AβO-induced reductions in proteasome activity in hippocampal synaptic fractions (Fig. 4h), suggesting that elevations in cAMP counteract proteasomal inhibition by AβOs.

AβOs are known to bind to synapses and trigger aberrant signaling in neurons^26–28^. Consistent with synaptic targeting by AβOs, exposure of isolated hippocampal synaptosomes to AβOs induced the inhibition of chymotrypsin-, trypsin- and caspase-like proteasome activities (Fig. 4i–k and Supplementary Fig. 1g–i). Control experiments revealed that preparations made from scrambled Aβ, which contains the same amino acid composition as Aβ but with a scrambled primary sequence, did not inhibit proteasome activity in synaptosomes (Supplementary Fig. 2). Similarly, treatment of synaptosomes with albumin, used as a non-AD-related protein negative control, had no effect on synaptosomal proteasome activity (Supplementary Fig. 2). These results suggest that synapse proteasome inhibition is specifically caused by AβOs and not likely a result of generic proteotoxicity.

The inhibition of proteasomes at synapses could be a result of AβO internalization and their direct association with proteasomes. To determine whether AβOs were internalized by synaptosomes, we exposed mouse synaptosomes to biotinylated AβOs for 20 min and subsequently incubated the synaptosomes in physiological or hypotonic buffer to lyse membranes. Consistent with previous findings by us and others indicating membrane localization of AβOs^27–29^, we detected AβOs in intact synaptosomes, but not in soluble fractions from lysed synaptosomes (Supplementary Fig. 3). Collectively, these results demonstrate that synaptic targeting of AβOs causes proteasome inhibition.

### Proteasome inhibition in APP/PS1 hippocampal synaptosomes

**Fig. 5:**
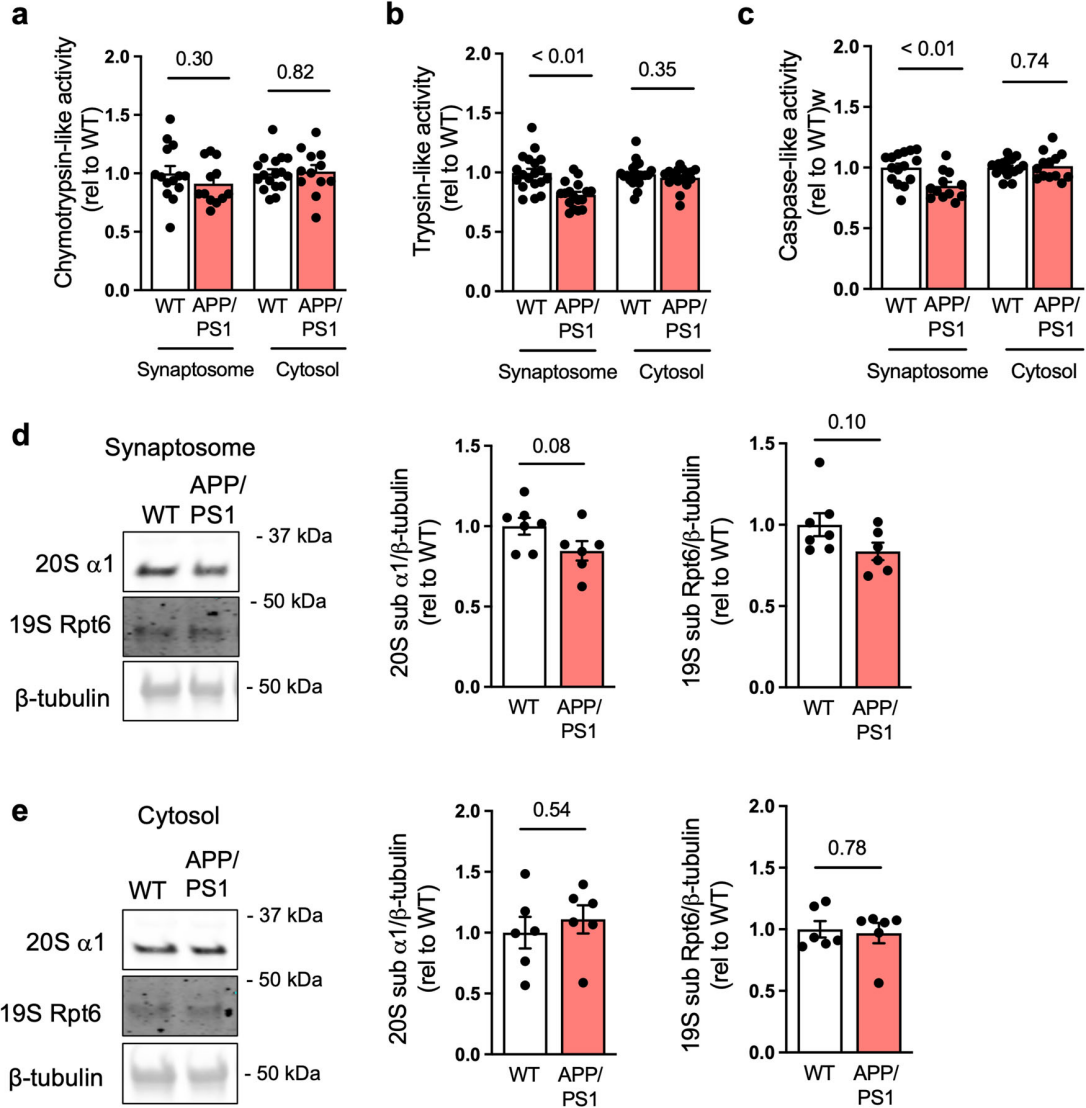
Reduced synaptic proteasome activity in the APP/PS1 hippocampus. Hippocampi from 12-month-old APP/PS1 mice or WT littermates were harvested for isolation of synaptosomes. **a**–**c** Proteasomal chymotrypsin-, trypsin-, and caspase-like activities were measured (chymotrypsin: *n* = 14 WT and 12 APP/PS1 for synaptosome, *n* = 17 WT and 12 APP/PS1 for cytosol; trypsin: 19 WT synaptosome, 17 APP/PS1 synaptosome, 21 WT cytosol, 17 APP/PS1 cytosol; caspase-like: 15 WT synaptosome, 12 APP/PS1 synaptosome, 17 WT cytosol, 13 APP/PS1 cytosol); two-tailed unpaired Student’s *t* test). **d**, **e** Proteasome 20S subunit α1 and 19S subunit Rpt6 were determined by Western blotting in synaptosome (**d**) (*n* = 7 WT and 6 APP/PS1; two-tailed unpaired Student’s *t* test) or cytosolic fractions (**e**) (*n* = 6 for all; two-tailed unpaired Student’s *t* test). Data are presented as mean ± SEM.

Next, we assessed proteasome activity in synaptosomes isolated from the hippocampi of 12-month-old APPswe/PS1dE9 mice (henceforth denoted APP/PS1 mice), a transgenic mouse model of AD that exhibits age-dependent brain accumulation of Aβ and memory deficits^30^. We found reduced trypsin- and caspase-like proteasome activities in synaptosomes from APP/PS1 mice compared to synaptosomes from the hippocampi of wild-type littermates (Fig. 5a–c and Supplementary Fig. 1j–l). Consistent with findings described above, no changes in proteasome activity were observed in the hippocampal cytosolic fraction. We further observed a trend of decrease in contents of 20S α1 and 19S Rpt6 proteins in APP/PS1 hippocampal synaptosomes, but not in the cytosolic fraction (Fig. 5d, e).

### Proteasome inhibition triggers neuronal oxidative stress, loss of dendritic spines, and impairs hippocampal protein synthesis

**Fig. 6:**
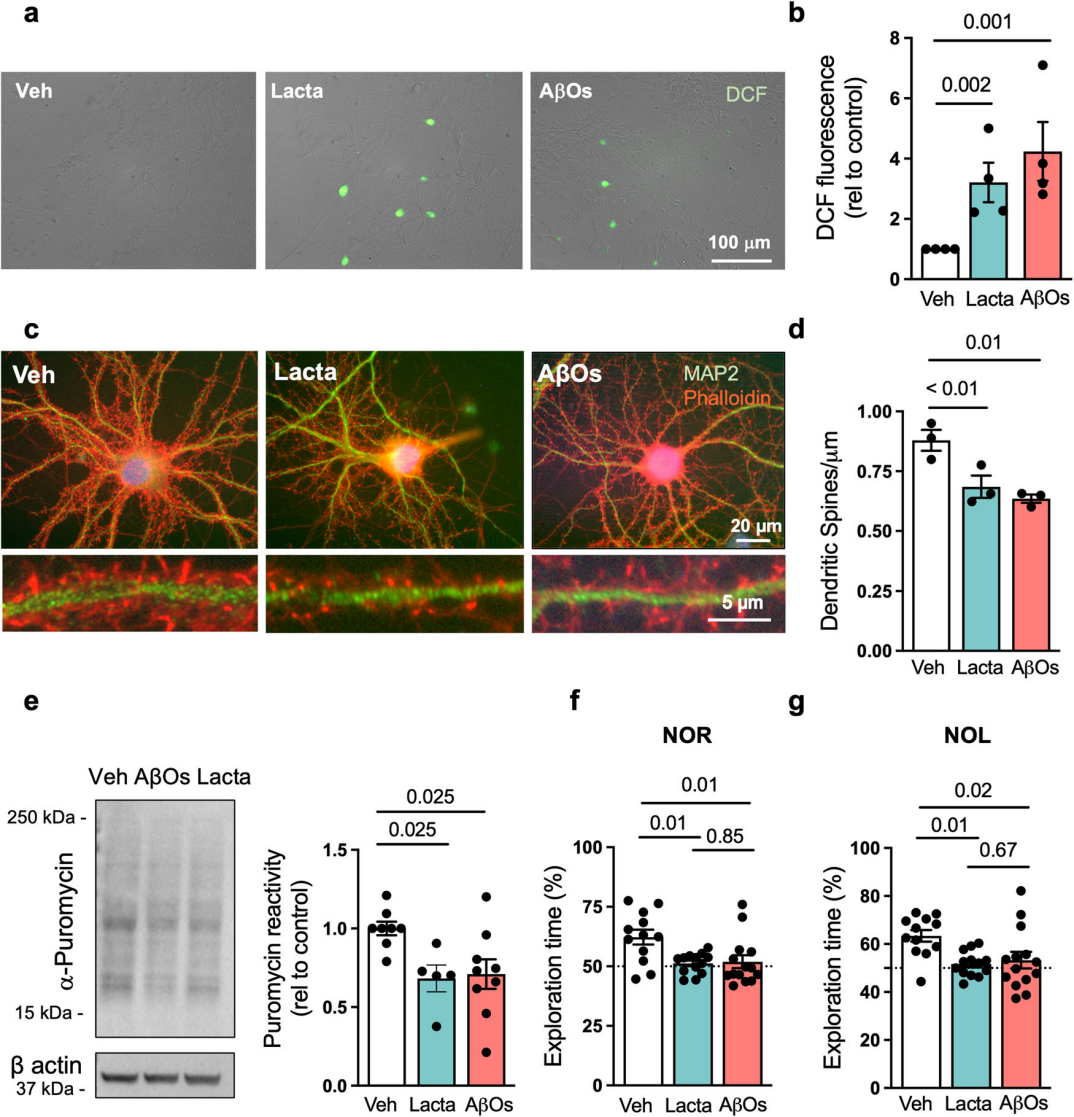
Proteasome inhibition triggers AD-like features in neurons. **a**, **b** Primary hippocampal cultures were exposed to vehicle, 0.5 µM AβOs or 0.5 µM lactacystin for 3 h and ROS were detected by DCF fluorescence (*n* = 4 independent cultures). Representative images in (**a**) show DCF fluorescence merged with brightfield images of cultures. Scale bar = 100 μm. **c**, **d** Primary hippocampal cultures were exposed to vehicle, 0.5 µM AβOs, or 0.5 µM lactacystin, and cells were double-labeled with neuronal marker MAP-2 (green) and F-actin probe phalloidin (red) for visualization of dendritic spines (*n* = 3 independent cultures). Images below the main panels are digital zoom images of selected dendrite segments. **e** 3-month-old C57/BL6 mice received intracerebroventricular infusions of vehicle, AβOs (10 pmol) or lactacystin (100 pmol). Hippocampi were harvested after 7 days, sliced, allowed to recover and incubated with puromycin for 45 min as described in “Methods”. SUnSET was performed by anti-puromycin immunolabeling (*n* = 8 mice for vehicle, 9 for AβOs; 5 mice for lactacystin). **f**, **g** 3-month-old mice received i.c.v. infusions of vehicle, AβOs (10 pmol) or lactacystin (100 pmol). Seven days after infusion, mice were tested in the novel object recognition (**e**) and novel object location (**f**) memory paradigms (*n* = 12 mice for vehicle, 13 mice for lactacystin, and 14 mice for AβOs). Symbols represent percentages of time of exploration of the novel object (or object at novel location) for individual mice. The dotted line at 50% corresponds to chance level. Unpaired two-tailed one-way ANOVA with Holm-Sidak post hoc test. Data are presented as mean ± SEM.

To determine whether proteasome inhibition could trigger pathological mechanisms that are characteristic of AD and deleterious to neuronal function and memory, we exposed primary hippocampal cultures in parallel to lactacystin, a pharmacological inhibitor of the proteasome, or to AβOs for 24 h. In line with previous reports^29,31,32^, exposure to AβOs triggered neuronal oxidative stress, as indicated by increased dichlorofluorescein (DCF) fluorescence, a readout of reactive oxygen species (ROS) accumulation (Fig. 6a, b). Proteasome inhibition by lactacystin elicited similar neuronal oxidative stress (Fig. 6a, b). Significantly, we further observed that treatment of hippocampal neurons with lactacystin resulted in dendritic spine loss, similar to neurons exposed to AβOs (Fig. 6c, d).

Accumulating evidence indicates that defective brain protein synthesis is implicated in memory impairments in AD^33–35^. This prompted us to investigate whether proteasome inhibition could impact brain protein synthesis. We assessed de novo protein synthesis using surface sensing of translation (SUnSET), a non-radioactive metabolic labeling method based on puromycin incorporation^36^, in hippocampal slices obtained 7 days after mice received a single i.c.v. infusion of lactacystin, AβOs or vehicle. Comparable reductions in protein synthesis were verified in hippocampal slices from mice treated with AβOs or lactacystin (Fig. 6e).

### Proteasome inhibition impairs object recognition and location memory in mice

Finally, to determine whether proteasome inhibition could result in memory impairments in mice, we assessed memory performance 7 days after mice received i.c.v. infusions of lactacystin or AβOs. I.c.v. infusion of lactacystin impaired both object recognition and object location memories, as indicated by reduced exploration times in the novel object recognition (NOR) and novel object location (NOL) tests (Fig. 6f, g), similar to memory impairments caused by AβOs (Fig. 6f, g). Taken together, these results demonstrate that proteasome inhibition recapitulates molecular and cognitive changes that develop in AD.

## Discussion

Defective brain proteostasis has been implicated as a core feature in the pathogenesis of neurodegenerative disorders, including AD^37,38^. Brains of AD patients and mouse models of AD exhibit altered control of protein synthesis^33,34^ and turnover^39,40^. A growing body of evidence indicates that defects in proteostasis are causally linked to cognitive failure in AD^33–35,41,42^, and that restoring protein homeostasis may be therapeutically relevant^33,43,44^.

While previous studies reported that disease-associated protein oligomers, including AβOs, inhibit isolated 20S proteasomes in vitro^45^ and that overexpression of mutant APP impairs brain proteasomal function in fly and mice^43^, whether AβOs selectively cause synaptic proteasome inhibition in AD remained to be further clarified. Building on early evidence that proteasome activity is inhibited in AD brains^13,14^ and on our finding of significantly increased levels of ubiquitinylated proteins in *post-mortem* AD brains, we show in several experimental models that AβOs selectively impair proteasome activity and reduce its localization at synapses, providing a mechanistic basis for AD-associated proteasome inhibition.

Several studies, including some by our group, have demonstrated that AβOs target synaptic sites in neurons^27–29^. Mechanistically, AβOs appear to initially bind to more diffuse sites in neuronal membranes but rapidly cluster onto synapses^28^ to impair signaling pathways^46^ and trigger synapse damage^11^. Furthermore, the synaptic presence of AβOs associates with cognitive impairment in the human brain^47^. Therefore, we hypothesized that aberrant signaling pathways mediating AβO-induced proteasome dysfunction could initiate at synapses. This was supported by our results showing that exposure of isolated synaptosome preparations to AβOs, but not to ScrAβ or albumin, is sufficient to trigger synaptic proteasome inhibition. We further note that, while a previous study demonstrated that AβOs can directly bind and inhibit the proteasome gate^45^, AβOs were not detected in the internal compartment of synaptosomes in our experimental conditions, suggesting that a direct interaction with Aβ is not the cause of synaptic proteasome inhibition in our study.

Evidence indicates that brain cAMP signaling is reduced by Aβ^48^, and that the cAMP/PKA pathway stimulates brain proteasome activity^25,49^. Mechanistically, elevating cAMP causes PKA-mediated phosphorylation of the 26S proteasome non-ATPase regulatory subunit 11 (Rpn6) to facilitate protein degradation^49^. Increased Rpn6 phosphorylation presumably triggers structural changes that promote 20S gate opening for substrate degradation^50,51^. Our current results showing that rolipram blocks the inhibition of synaptic proteasome activity by AβOs support the notion that impaired cAMP signaling might contribute to AβO-induced changes in proteasome function and regulation. While additional studies are required to further elucidate the mechanisms underlying proteasome inhibition in AD, our data support a model in which AβOs instigate aberrant signaling pathways culminating in proteasome inhibition rather than a direct interaction of AβOs with proteasomes.

Our finding of AβO-induced proteasome mislocalization suggested that intracellular trafficking of proteasomes to/from synapses may be impaired in AD. This was further supported by results showing that dynein inhibition prevented synaptic proteasome mislocalization in hippocampal cultures. Dynein motors have been described as essential for efficient axonal and dendritic cargo transport^52^, and previous studies by our group and others have demonstrated that AβOs impair molecular motor function and disrupt microtubule-dependent organelle transport in neurons^29,53–55^. Therefore, it seems plausible that AβO-dependent changes in cargo transport may contribute to proteasome mislocalization in hippocampal neurons.

Collectively, our findings indicate that a combination of reduced local proteasome activity and proteasome mislocalization from synapses to non-synaptic compartments may converge to cause neuronal dysfunction in AD. Considering that activity-dependent recruitment of proteasomes is essential for synaptic structural plasticity, impaired proteasome activity at synapses likely contributes to impaired synapse function and may render neurons vulnerable to toxic insults.

In addition to their role in the degradation of misfolded or damaged proteins, novel assemblies and functions have been proposed for proteasomes in neurons. A plasma membrane-associated 20S particle, termed neuroproteasome, was shown to mediate the export of neuroactive peptides at synapses^56^ and to regulate neural circuitry and behavior^57^. Further, recent results indicate that 19S particles regulate synaptic function by modulating the synaptic availability of AMPA glutamate receptors independently of proteasome catalytic activity^58^. Therefore, the reduction in proteasome content at synapses induced by AβOs may be detrimental by alternative mechanisms in addition to its classical function in protein degradation.

Our results indicate that pharmacological inhibition of proteasome activity by lactacystin is sufficient to trigger molecular, cellular, and cognitive alterations that are hallmark features of AD, including neuronal oxidative stress, impaired mRNA translation, loss of dendritic spines, and memory defects. This suggests that altered proteasome function can be an underlying factor in AD pathogenesis. In agreement with this notion, previous reports have demonstrated that proteasome inhibition causes behavioral alterations^59^ and that increasing proteasome activity rescues some disease phenotypes in AD models^24,25,43^.

AD is a progressive disorder characterized by brain accumulation of aggregated forms of Aβ, including AβOs. In sporadic AD, it is thought that the buildup of Aβ results largely from impaired clearance from the brain^60^. Hence, impaired proteasome localization/function and Aβ accumulation may converge to compromise neuronal function and memory in AD. Significantly, we detected a selective inhibition of synaptic proteasomes in the hippocampi of 12-month-old APP/PS1 mice, which develop age-associated Aβ accumulation and memory impairment^30^. These findings support the notion that synaptic proteasome dysfunction develops upon chronic amyloid pathology.

In summary, current results link synaptic proteasome inhibition and mislocalization to the pathogenesis of AD, and indicate that the synaptic proteasome pool is vulnerable to inhibition in AD. Our findings further reveal that brain proteasome inhibition is associated with AD neuropathological hallmarks and memory impairment, suggesting that development of pharmacological approaches to preserve or enhance brain proteasome function may delay the onset or be therapeutically relevant in AD.

## Materials and methods

### Postmortem human brain tissue

Hippocampal samples from AD or non-cognitively impaired individuals (controls) were obtained from the Brain Bank of the Brazilian Aging Brain Study Group, School of Medicine of the University of Sao Paulo (IRB protocol 49903421.4.0000.5257). Brain samples were obtained following written consent, and collection was approved by the Ethics Committee of the University of Sao Paulo. Diagnosis of AD was confirmed by histopathological detection of neurofibrillary tangles and amyloid plaques, and by clinical dementia rating (CDR) scores derived from interviews with a proximal family member or the donor caregiver^61,62^. The control group consisted of cases with CDR = 0, and the AD group included cases with CDR ranging from 1 to 3.

### Human cortical slices

Human cortical tissue was obtained from the temporal lobe of adult patients with drug-refractory epilepsy who were subjected to surgery for removal of the hippocampal epileptic focus, as previously described^63,64^. Experiments involving human cortical tissue were approved by the Committee for Research Ethics of the Clementino Fraga Filho University Hospital of the Federal University of Rio de Janeiro (protocol 0069.0.197.000-05). Donors gave written informed consent for the use of brain tissue that would otherwise have been discarded. Cortical tissue was obtained and sectioned at 400 µm using a McIlwain chopper. Slices were plated in 6 well-plates with Neurobasal A medium containing 2% B27 (Gibco), 500 µM glutamine, 5 ng/ml FGF2, 2 µM DHEA, 1 ng/ml BDNF and 50 µg/ml gentamycin (Gibco), as described^63,65^. The slices were kept in culture for 7 days at 37 °C at 5% CO_2_, were exposed to 0.5 µM AβOs or vehicle for 24 h and were then processed for biochemical analysis.

### Animals

Male Swiss mice were obtained from the animal facility at the Federal University of Rio de Janeiro and were housed in groups of 3–5 mice per cage, with food and water ad libitum, in temperature and humidity-controlled rooms, on a 12 h light/dark cycle. Male and female APPswe/PS1dE9 mice or wild-type (WT) littermates on a C57BL/6J background were obtained from the Jackson Laboratories and were bred at our facility. All procedures were approved by the Committee for Use of Animals in Research of the Center for Health Sciences at the Federal University of Rio de Janeiro (IACUC protocol 137/15).

### Synaptosome preparation

Hippocampal tissue was dissociated for synaptosome purification using the Synaptic Protein Extraction Reagent (Syn-PER; Thermo Fisher; #87793), as recommended by the manufacturer. Briefly, samples were homogenized in Syn-PER reagent, centrifuged at 1200 × *g* for 10 min at 4 °C and the supernatant was collected for another centrifugation at 15,000 × *g* for 20 min at 4 °C. The pellet (synaptic fraction) was resuspended in Syn-PER reagent and used for the experiments. Experiments also included the cytosolic (non-synaptic) fraction for comparison with the synaptic fraction. In some experiments, AβOs or a preparation made from scrambled Aβ following the exact same procedure as utilized to make AβOs were added at 1 μM directly to isolated synaptosome preparations for 1 h. For AβO detection in synaptosomes, synaptosomal preparations were treated with vehicle or biotinylated AβOs (Echelon Biosciences, 1 μM) for 20 min at 37 °C. The preparations were either homogenized with RIPA buffer or with a hypotonic buffer (10 mM Tris-HCl, pH 7.5) for 30 min on ice. The hypotonic buffer homogenate was then centrifuged at 15,000 × *g* for 10 min at 4 °C. The supernatant was collected for Western blotting, and both homogenates were probed for fluorophore-conjugated streptavidin (1:5000, LiCor) and developed on Odyssey (LiCor).

### Western blotting

Tissue was dissociated by sonication in RIPA buffer (Thermo Fisher; #89900) containing protease and phosphatase inhibitors (Thermo Fisher), centrifuged for 10 min at 10,000 × *g* at 4 °C, and the supernatant was collected for further analysis. Protein concentration was determined using the BCA Kit (Thermo Fisher; #23222) and samples were prepared to a final concentration of 2 μg/μl. After boiling samples for 10 min, 20 μg total protein were loaded per lane and resolved in 4–20% acrylamide gradient Tris-glycine gels (Biorad; #4561096). Proteins were then electrotransferred to a nitrocellulose membrane at 300 mA for 60 min at 4 °C. Primary antibodies used were: ubiquitin (1:1000; Cell Signaling; #3933S), GAPDH (1:20,000; Abcam, #ab9484), 20S subunit α1 (1:500; Abcam; #ab3325), 19S subunit Rpt6 (1:500; Enzo Life Sciences; #BML-PW9265), β-tubulin (1:20,000; Abcam; #ab15568), β-actin (1:20,000; Abcam; #ab6276), puromycin (clone 12D10, 1:1000; EMD Millipore). Immunoblots were developed using IR dye-conjugated fluorescent secondary antibodies (1:5000, LiCor). Quantification was performed using Fiji/ImageJ^66^. Full blots are presented in Supplementary Figs. 4 and 5.

### Hippocampal cell culture, immunocytochemistry, and phalloidin labeling

Cells were harvested from hippocampi of E18 rats and maintained in Neurobasal medium (Thermo Fisher) supplemented with antibiotics and B27 (Thermo Fisher), as described^26,35,53^. Cells were treated at 17-18 DIV with vehicle, 0.5 µM AβOs or 0.5 µM lactacystin for 24 h and then fixed for 10 min with 4% paraformaldehyde for immunocytochemistry as described^35^. When present, erythro-9-[3-(2-hydroxynonyl)]adenine (EHNA; 100 µM; Sigma-Aldrich) was added 15 min before AβOs. Cells were labeled with anti-20S α1 (1:400; Abcam; ab3325), anti-19S Rpt6 (1:400; Enzo Life Sciences; #BML-PW9265), anti-MAP2 (Millipore; #ab5622) or Alexa 594-conjugated phalloidin (1:40; Sigma-Aldrich; #A12381) overnight at 4 °C, followed by Alexa-conjugated secondary antibodies (Invitrogen) for 2 h at room temperature. Experiments were replicated in 3–6 independent neuronal cultures (as indicated in figure legends). Images were acquired on a Zeiss AxioObserver Z1 microscope and were analyzed using Fiji/ImageJ software.

### AβO preparation, intracerebroventricular (i.c.v.) infusions and rolipram treatment

Aβ_1-42_ peptide was purchased from Echelon Biosciences. Aβ oligomerization was performed as previously described^26,65^. Oligomer preparations were stored at 4 °C, used within 72 h and were routinely characterized by high performance size-exclusion liquid chromatography (SEC-HPLC)^63,67,68^. I.c.v. infusions of AβOs (10 pmol) in mice were performed as described^67^. Briefly, mice were briefly (<2 min) anesthetized with 2.5% isoflurane and 3 μl of AβOs (10 pmol) (or an equivalent volume of vehicle) were injected 1 mm to the right of the midline point equidistant to each eye, and 1 mm posterior to a line drawn through the anterior base of the eyes^67^. After injection, the needle was kept in place for 3 s to prevent any backflow, and mice were returned to their cages for recovery. For experiments with rolipram (Sigma-Aldrich), mice were treated with saline or rolipram (0.1 mg/kg, subcutaneously) for 3 days prior to AβOs infusion, and continued receiving subcutaneous injections of rolipram or saline for 7 additional days before tissue collection. Mice were euthanized 40 min after the last s.c. injection.

### Proteasome activity

Cultured cells were scraped off the cell culture well and homogenized in buffer containing 40 mM Tris, pH 7.2, 50 mM NaCl, 2 mM β-mercaptoethanol, 2 mM ATP, 5 mM MgCl_2_, 1 mM EDTA. Human or mouse brain tissue were homogenized in a glass tissue grinder in buffer containing 20 mM Tris, pH 7.5, and 5 mM MgCl_2_. Protein concentration was determined by BCA and samples (50 µg protein) were assayed in a fluorogenic proteasome activity assay (UBPBio; #J4120), following manufacturer’s instructions.

### Protein synthesis determination using SUnSET in hippocampal slices

A non-radioactive method for detection of newly synthesized polypeptides was used^69^. Three-month-old mice received an i.c.v. infusion of vehicle, 10 pmol AβOs or 100 pmol lactacystin, and hippocampi were collected 7 days later. Hippocampal slices (400 μm) were prepared and allowed to recover in aCSF for 2 h at 30 °C. Slices were then exposed to puromycin (5 µg/ml; 45 min) and processed for Western blotting. Blots were developed using anti-puromycin primary antibody, and puromycin incorporation into newly synthesized proteins was evaluated. β-actin was used as loading control.

### Novel object recognition (NOR) and novel object location (NOL) memory tests

Before the tests, animals were allowed to explore an open field arena (0.3 × 0.3 × 0.45 m^3^) for 5 min and locomotor activity was assessed by measurements of distance traveled and velocity. In the novel object recognition (NOR) training session, mice were exposed to two identical objects for 5 min, and the amount of time spent exploring each object was measured. One hour later, mice were re-exposed to the open field arena for 5 min during the test session, in which one of the previously used (familiar) objects were replaced by a novel object. Time exploring the familiar and novel objects were measured. Twenty-four hours later, mice were exposed again to the open field arena for novel object location (NOL) assessment, when one of the objects used in the training session was displaced. Time exploring each object was scored. Animals were excluded from the analyses if they had less than 5 s of total exploration in the training or the testing phase.

### Reactive oxygen species (ROS)

ROS accumulation was evaluated in primary rat hippocampal neuronal cultures using CM-H_2_DCFDA (Thermo Fisher), a fluorescent probe sensitive to ROS, as described^31,32,70^. Briefly, CM-H_2_DCFDA (2 μM) was added to the cultures for 40 min in a CO_2_ incubator, the wells were washed 3 times with warm (37 °C) PBS, and cells were maintained in neurobasal medium without phenol red during acquisition. Neurons were immediately imaged on a Nikon inverted microscope for fluorescence and bright field acquisition. Representative images shown are merged images of DCF fluorescence and bright field. Analysis of DCF fluorescence data was carried out using Fiji/ImageJ.

### Statistics and reproducibility

Data are expressed as means ± S.E.M. and were analyzed using GraphPad Prism 8 software. Sample size for each experiment was estimated by previous experience with different experiments. No algorithm or software was used to randomize animal subjects. Animals were randomly assigned to groups by trained researchers performing each experiment. When comparing four experimental groups, two-way ANOVA was performed, followed by appropriate post hoc tests, as stated in “Figure Legends”. Values of *p* ≤ 0.05 were considered statistically significant.

### Reporting summary

Further information on research design is available in the Nature Portfolio Reporting Summary linked to this article.

### Supplementary information

Supplementary Material

Supplementary Data 1

Reporting Summary

## Data Availability

The data that support the findings of this study are available from the corresponding author upon reasonable request. Data used in the main figures are available as Supplementary Data 1.
