# Supplementary material for: Synaptic proteasome is inhibited in Alzheimer’s disease models and associates with memory impairment in mice
Source: Commun Biol. 2023 Nov 7;6:1127. doi: 10.1038/s42003-023-05511-9 (PMC10630330; doi:10.1038/s42003-023-05511-9)
Supplement: Supplementary file 1 — Supplementary Material [file 42003_2023_5511_MOESM1_ESM.pdf]

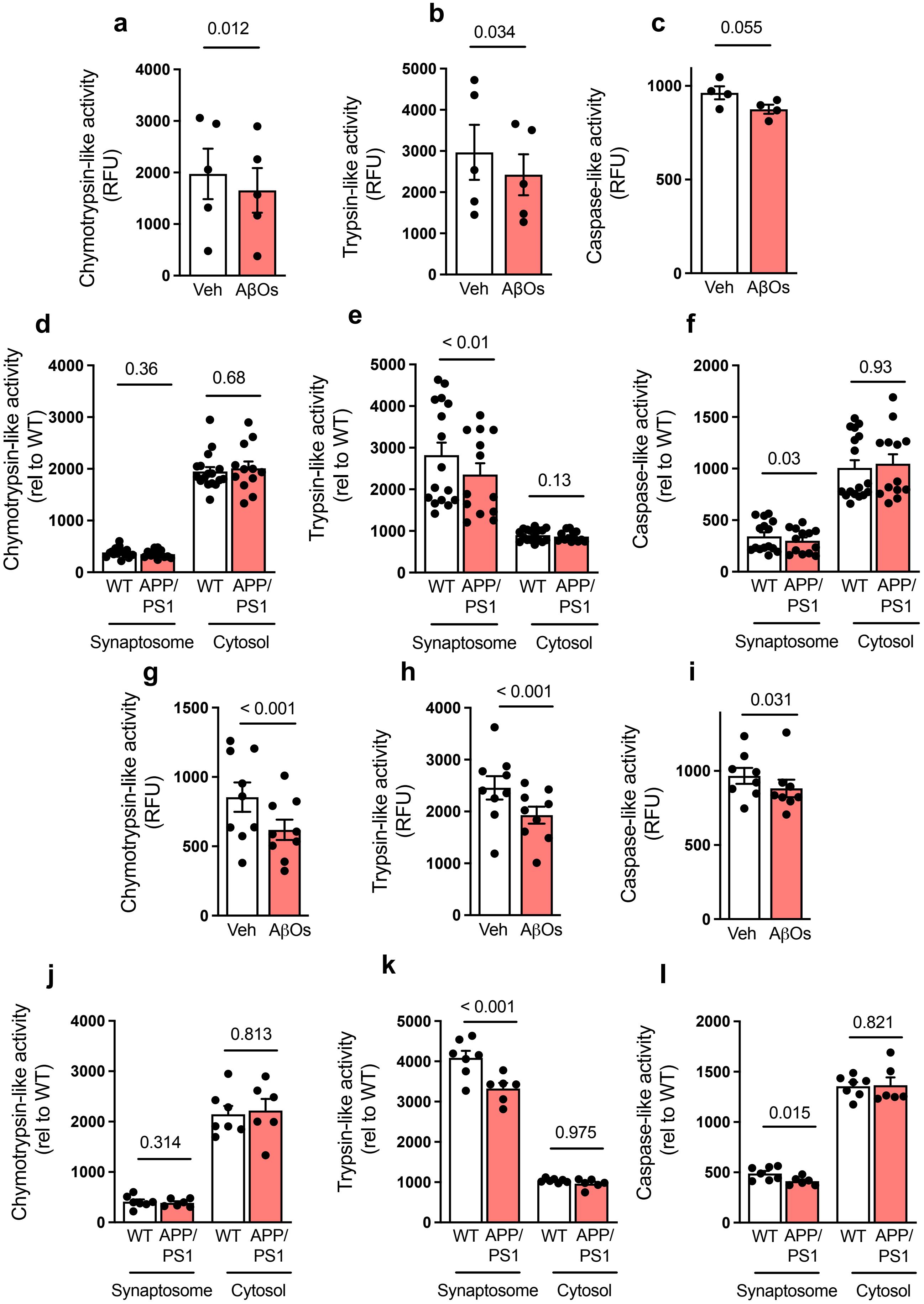
a
0.012
Chymotrypsin-like activity (RFU)
Veh
AβOs
b
0.034
Trypsin-like activity (RFU)
c
0.055
Caspase-like activity (RFU)
d
0.36
0.68
Chymotrypsin-like activity (rel to WT)
WT
APP/PS1
Synaptosome
Cytosol
e
< 0.01
0.13
Trypsin-like activity (rel to WT)
f
0.03
0.93
Caspase-like activity (rel to WT)
g
< 0.001
h
< 0.001
i
0.031
j
0.314
0.813
k
< 0.001
0.975
l
0.015
0.821

Supplementary Figure 1. Relative fluorescence units related to main figures in the manuscript . (A-L) Data expressed as relative fluorescence units for Panels 2A-C (A-C), 3A-C (D-F), 3I-K (G-I), 4A-C (J-L).

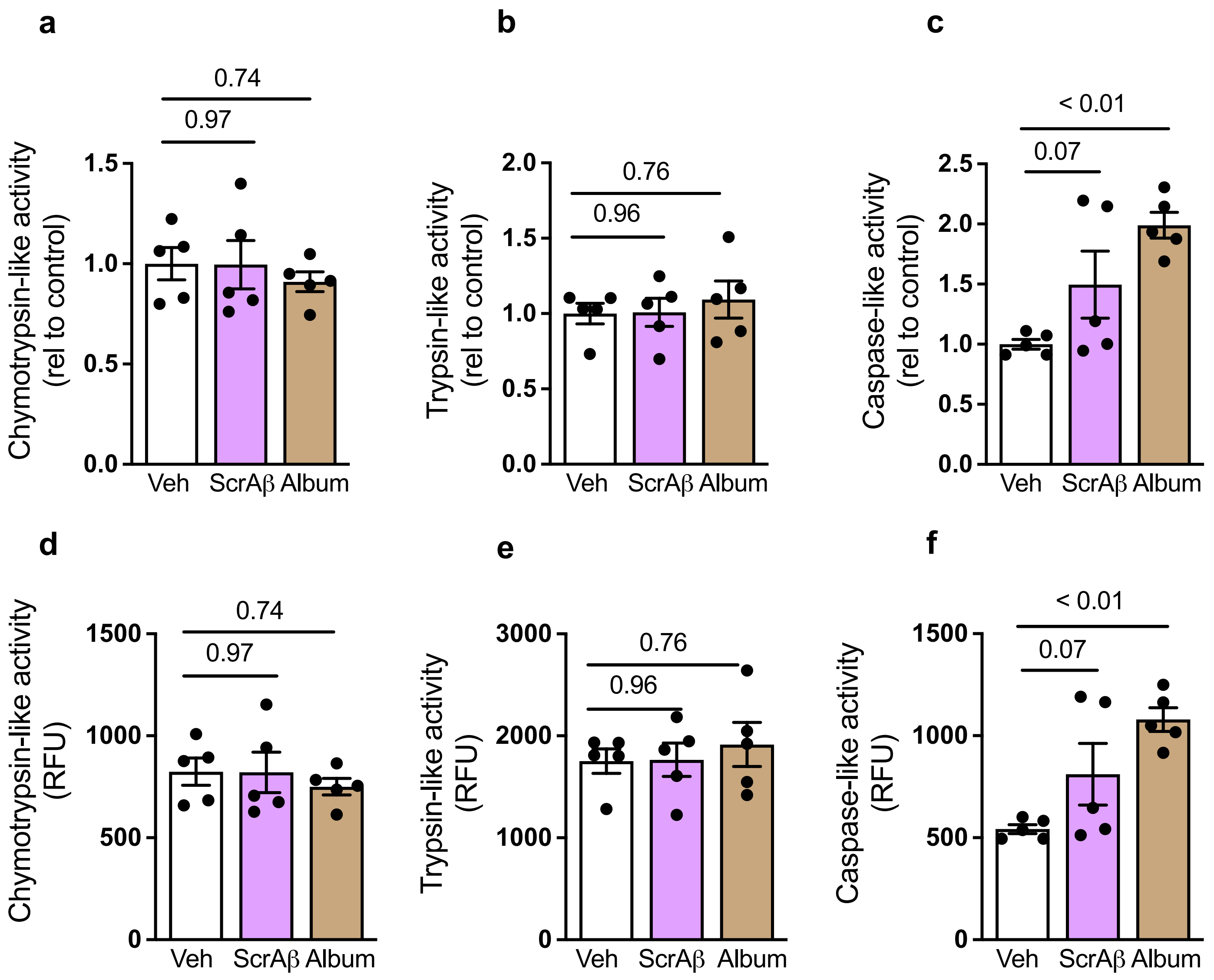
a
0.74
0.97
Chymotrypsin-like activity (rel to control)
1.5
1.0
0.5
0.0
Veh
ScrAβ
Album
b
0.76
0.96
Trypsin-like activity (rel to control)
2.0
1.5
1.0
0.5
0.0
Veh
ScrAβ
Album
c
< 0.01
0.07
Caspase-like activity (rel to control)
2.5
2.0
1.5
1.0
0.5
0.0
Veh
ScrAβ
Album
d
0.74
0.97
Chymotrypsin-like activity (RFU)
1500
1000
500
0
Veh
ScrAβ
Album
e
0.76
0.96
Trypsin-like activity (RFU)
3000
2000
1000
0
Veh
ScrAβ
Album
f
< 0.01
0.07
Caspase-like activity (RFU)
1500
1000
500
0
Veh
ScrAβ
Album

Supplementary Figure 2. Scrambled Aβ peptide or albumin does not cause proteasome inhibition. (A-C) Hippocampi from naive mice were harvested and synaptosomes were isolated. Synaptosome preparations were then exposed to Scrambled Aβ (ScrAβ), albumin, or vehicle for 1 h at 37 ºC, and proteasomal chymotrypsin-, trypsin-, and caspase-like activities were measured and normalized by control. (D-F) Data expressed as relative fluorescence units for graphs A-C ($n = 5$ synaptosomal preparations from independent mice; Two-tailed One-way Anova with Holm Sidak correction.

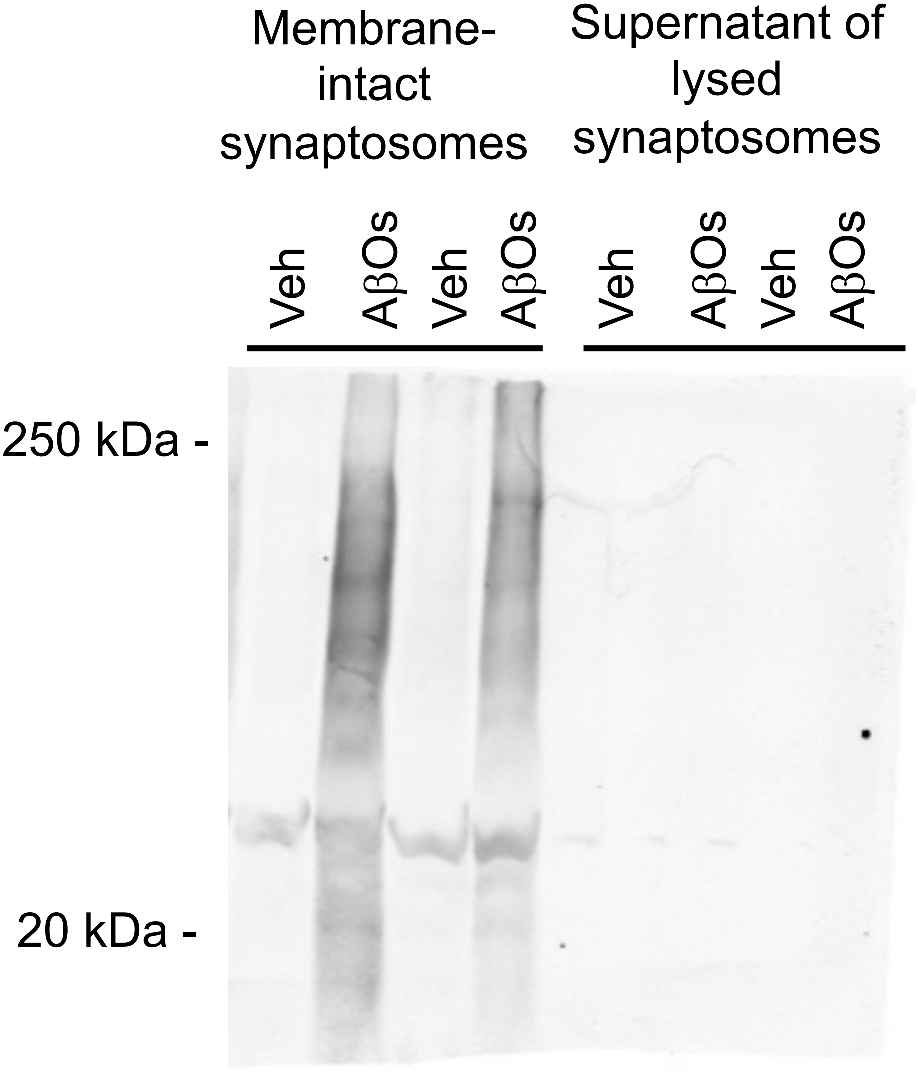
Membrane-intact synaptosomes
Supernatant of lysed synaptosomes
Veh
AβOs
Veh
AβOs
Veh
AβOs
Veh
AβOs
250 kDa -
20 kDa -

Supplementary Figure 3. AβOs were not detected in soluble fractions of synaptosome preparations. Synthetic AβOs or vehicle were added to synaptosome preparations for 20 min at 37º C. The synaptosome preparations were either lysed in standard conditions or with a hypothonic buffer and centrifuged to 15 000 g for 10 min at 4ºC to remove membrane fraction. We detected AβOs selectively in total synaptosomes preparations and not in the lysed supernatant, nor in vehicle-treated synaptosomes (n = 2).

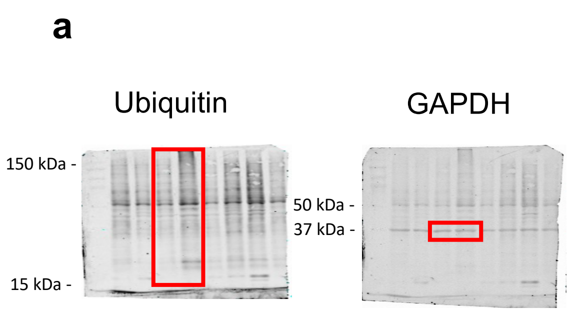

Fig 1a

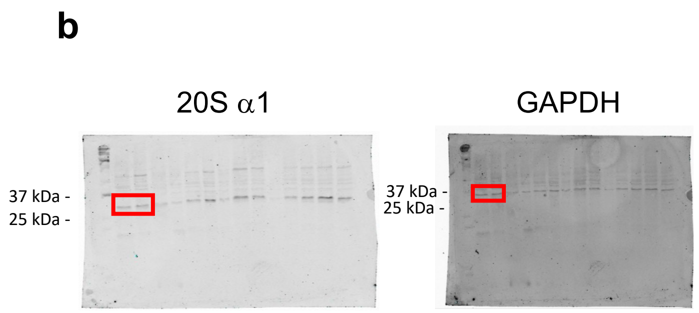

Fig 1b

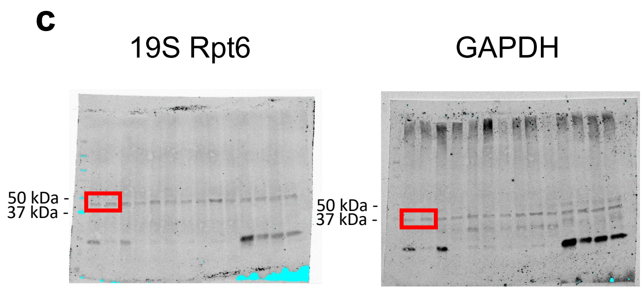

Fig 1c

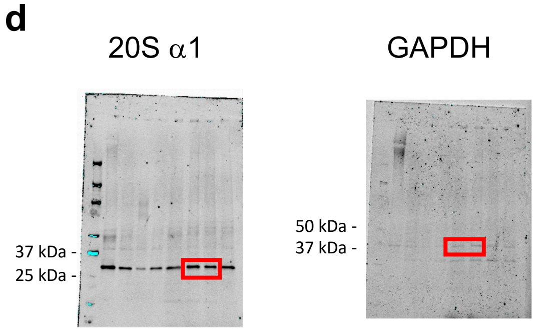

Fig 1g

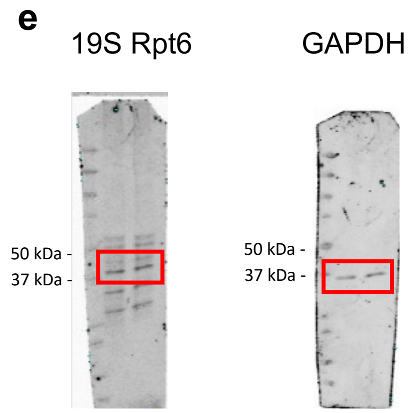

Fig 1h

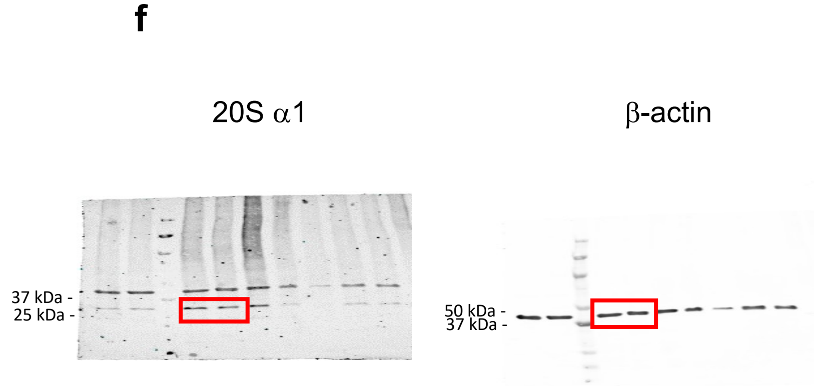

Fig 2d

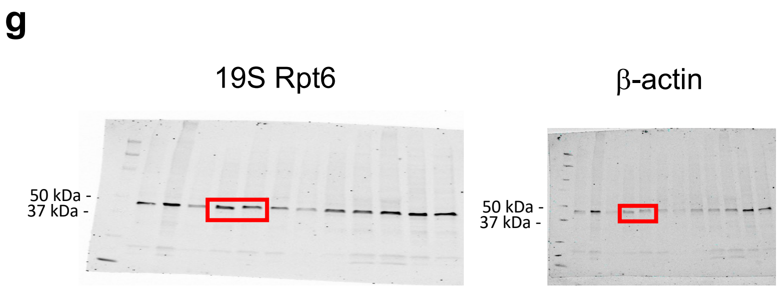

Fig 2e

Supplementary Figure 4. Full blots relative to panels presented in Figures 1 and 2.

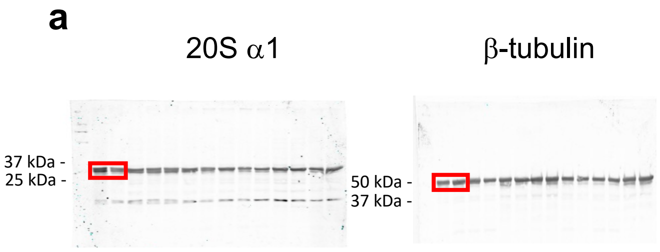

Fig 4d

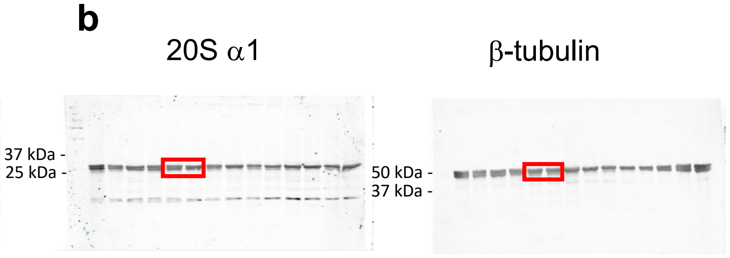

Fig 4e

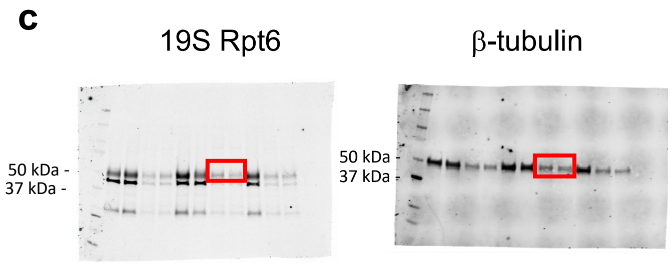

Fig 4f

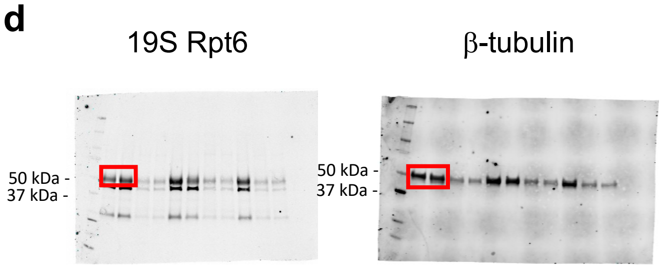

Fig 4g

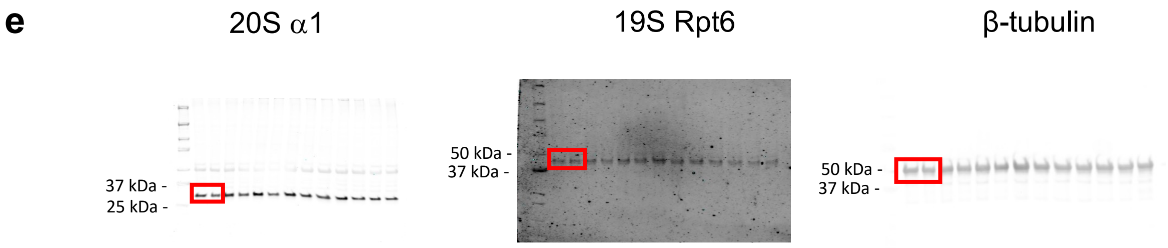

Fig 5d

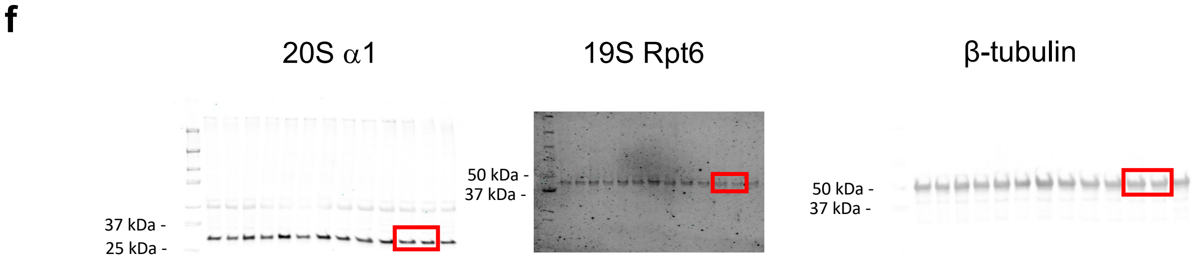

Fig 5e

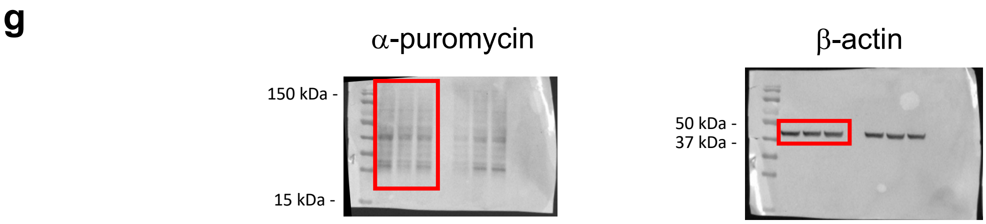

Fig 6e

Supplementary Figure 5. Full blots relative to panels presented in Figures 4, 5 and 6.
