# Supplementary material for: Synaptic proteasome is inhibited in Alzheimer’s disease models and associates with memory impairment in mice
Source: Commun Biol. 2023 Nov 7;6:1127. doi: 10.1038/s42003-023-05511-9 (PMC10630330; doi:10.1038/s42003-023-05511-9)
Supplement: Supplementary file 3 — Reporting Summary [file 42003_2023_5511_MOESM3_ESM.pdf]

Corresponding author(s): Sergio T. Ferreira

Last updated by author(s): Oct 15, 2023

Reporting Summary

Nature Portfolio wishes to improve the reproducibility of the work that we publish. This form provides structure for consistency and transparency in reporting. For further information on Nature Portfolio policies, see our Editorial Policies and the Editorial Policy Checklist.

Statistics

For all statistical analyses, confirm that the following items are present in the figure legend, table legend, main text, or Methods section.

n/a	Confirmed	
☐	☐	The exact sample size (n) for each experimental group/condition, given as a discrete number and unit of measurement
☐	☒	A statement on whether measurements were taken from distinct samples or whether the same sample was measured repeatedly
☐	☒	The statistical test(s) used AND whether they are one- or two-sided<br>*Only common tests should be described solely by name; describe more complex techniques in the Methods section.*
☒	☐	A description of all covariates tested
☐	☒	A description of any assumptions or corrections, such as tests of normality and adjustment for multiple comparisons
☐	☒	A full description of the statistical parameters including central tendency (e.g. means) or other basic estimates (e.g. regression coefficient) AND variation (e.g. standard deviation) or associated estimates of uncertainty (e.g. confidence intervals)
☐	☒	For null hypothesis testing, the test statistic (e.g. F, t, r) with confidence intervals, effect sizes, degrees of freedom and P value noted<br>*Give P values as exact values whenever suitable.*
☒	☐	For Bayesian analysis, information on the choice of priors and Markov chain Monte Carlo settings
☒	☐	For hierarchical and complex designs, identification of the appropriate level for tests and full reporting of outcomes
☒	☐	Estimates of effect sizes (e.g. Cohen's d, Pearson's r), indicating how they were calculated

Our web collection on statistics for biologists contains articles on many of the points above.

Software and code

Policy information about availability of computer code

Data collection	ANY-Maze 2, Zeiss Axiovision 4.8, Microsoft Office 2008 for Mac, Li-cor Image Studio 3.0, SpectaMax Softmax Pro 6.
Data analysis	ANY-Maze 2, Zeiss Axiovision 4.8, Microsoft Office 2008 for Mac, GraphPad Prism 6, Fiji 3.0.

For manuscripts utilizing custom algorithms or software that are central to the research but not yet described in published literature, software must be made available to editors and reviewers. We strongly encourage code deposition in a community repository (e.g. GitHub). See the Nature Portfolio guidelines for submitting code & software for further information.

Data

Policy information about availability of data

All manuscripts must include a data availability statement. This statement should provide the following information, where applicable:

- Accession codes, unique identifiers, or web links for publicly available datasets
- A description of any restrictions on data availability
- For clinical datasets or third party data, please ensure that the statement adheres to our policy

The data that support the findings of this study are available from the corresponding author upon reasonable request. Data used in the main figures are available as Supplementary Data in this manuscript.

Human research participants

Policy information about studies involving human research participants and Sex and Gender in Research.

Reporting on sex and gender	*Use the terms sex (biological attribute) and gender (shaped by social and cultural circumstances) carefully in order to avoid confusing both terms. Indicate if findings apply to only one sex or gender; describe whether sex and gender were considered in study design whether sex and/or gender was determined based on self-reporting or assigned and methods used. Provide in the source data disaggregated sex and gender data where this information has been collected, and consent has been obtained for sharing of individual-level data; provide overall numbers in this Reporting Summary. Please state if this information has not been collected. Report sex- and gender-based analyses where performed, justify reasons for lack of sex- and gender-based analysis.*
Population characteristics	*Describe the covariate-relevant population characteristics of the human research participants (e.g. age, genotypic information, past and current diagnosis and treatment categories). If you filled out the behavioural & social sciences study design questions and have nothing to add here, write "See above."*
Recruitment	*Describe how participants were recruited. Outline any potential self-selection bias or other biases that may be present and how these are likely to impact results.*
Ethics oversight	*Identify the organization(s) that approved the study protocol.*

Note that full information on the approval of the study protocol must also be provided in the manuscript.

Field-specific reporting

Please select the one below that is the best fit for your research. If you are not sure, read the appropriate sections before making your selection.

☒ Life sciences ☐ Behavioural & social sciences ☐ Ecological, evolutionary & environmental sciences

For a reference copy of the document with all sections, see nature.com/documents/nr-reporting-summary-flat.pdf

Life sciences study design

All studies must disclose on these points even when the disclosure is negative.

Sample size	Sample size for each experiment was estimated by previous experience with different experiments.
Data exclusions	In NOR/NOL tests, animals were excluded from the analyses if they had less than 5 s of total exploration in the training or the testing phase. No other exclusion criteria was employed in animal studies.
Replication	Studies involved replication of experimental findings whenever possible. Behavioral tests were performed in different cohorts of animals. Culture experiments were performed independent cultures.
Randomization	No algorithm or software was used to randomize animal subjects. Animals were randomly assigned to groups by trained researchers performing each experiment.
Blinding	Experiments involving image analyses and behavioral tests in mice were performed in blinded fashion.

Reporting for specific materials, systems and methods

We require information from authors about some types of materials, experimental systems and methods used in many studies. Here, indicate whether each material, system or method listed is relevant to your study. If you are not sure if a list item applies to your research, read the appropriate section before selecting a response.

Materials & experimental systems

n/a	Involved in the study	
☐	☒	Antibodies
☒	☐	Eukaryotic cell lines
☒	☐	Palaeontology and archaeology
☐	☒	Animals and other organisms
☒	☐	Clinical data
☒	☐	Dual use research of concern

Methods

n/a	Involved in the study	
☒	☐	ChIP-seq
☒	☐	Flow cytometry
☒	☐	MRI-based neuroimaging

Antibodies

Antibodies used	Primary antibodies used were: ubiquitin (1:1,000; Cell Signaling; #3933S), GAPDH (1:20,000; Abcam, #ab9484), 20S subunit α1 (1:500; Abcam; #ab3325), 19S subunit Rpt6 (1:500; Enzo Life Sciences; #BML-PW9265), -tubulin (1:20,000; Abcam; #ab15568), -actin (1:20,000; Abcam; #ab6276), puromycin (clone 12D10, 1:1000; EMD Millipore). Immunoblots were developed using IR dye-conjugated fluorescent secondary antibodies (1:5,000, LiCor).
Validation	Antibodies were validated and used according to manufacturer's instructions.

Animals and other research organisms

Policy information about studies involving animals; ARRIVE guidelines recommended for reporting animal research, and Sex and Gender in Research

Laboratory animals	This work involved the use of 3-month old male WT Swiss mice, as reported in each figure. In experiments involving transgenic APP/PS1 mice (on a C57BL/6 background), both males and females were used at the indicated ages.
Wild animals	N/A
Reporting on sex	Most of the studies were performed in male mice, but results on proteasome inhibition in APP/PS1 hippocampi were performed on both sexes with similar results.
Field-collected samples	N/A
Ethics oversight	All procedures involving animals were approved by the Committee for Use of Animals in Research of the Center for Health Sciences at the Federal University of Rio de Janeiro (IACUC protocol 137/15).

Note that full information on the approval of the study protocol must also be provided in the manuscript.
